# A Large Proportion of the Mexican Population Remained Susceptible to A(H1N1)pdm09 Infection One Year after the Emergence of 2009 Influenza Pandemic

**DOI:** 10.1371/journal.pone.0150428

**Published:** 2016-03-22

**Authors:** Vic Veguilla, Hugo López-Gatell, Irma López-Martínez, Rodrigo Aparicio-Antonio, Gisela Barrera-Badillo, Julieta Rojo-Medina, Felicia Liaini Gross, Stacie N. Jefferson, Jacqueline M. Katz, Mauricio Hernández-Ávila, Celia M. Alpuche-Aranda

**Affiliations:** 1 Influenza Division, National Center for Immunization and Respiratory Diseases, Centers for Disease Control and Prevention, Atlanta, Georgia, United States of America; 2 Dirección General Adjunta de Epidemiología, Secretaría de Salud, Mexico City, México; 3 Laboratorio de Virus Respiratorios, Departamento de Virología, Instituto de Diagnóstico y Referencia Epidemiológicos, Mexico City, México; 4 Centro Nacional de la Transfusión Sanguínea, Secretaría de Salud, Mexico City, México; 5 Subsecretaría de Prevención y Promoción de la Salud, Secretaría de Salud, Mexico City, México; 6 Dirección General Adjunta del Instituto de Diagnóstico y Referencia Epidemiológicos, Mexico City, México; US Food and Drug Administration, UNITED STATES

## Abstract

**Background:**

The 2009 H1N1 influenza pandemic initially affected Mexico from April 2009 to July 2010. By August 2010, a fourth of the population had received the monovalent vaccine against the pandemic virus (A(H1N1)pdm09). To assess the proportion of the Mexican population who remained potentially susceptible to infection throughout the summer of 2010, we estimated the population seroprevalence to A(H1N1)pdm09 in a serosurvey of blood donors.

**Methods:**

We evaluated baseline cross-reactivity to the pandemic strain and set the threshold for seropositivity using pre-pandemic (2005–2008) stored serum samples and sera from confirmed A(H1N1)pdm09 infected individuals. Between June and September 2010, a convenience sample serosurvey of adult blood donors, children, and adolescents was conducted in six states of Mexico. Sera were tested by the microneutralization (MN) and hemagglutination inhibition (HI) assays, and regarded seropositive if antibody titers were equal or exceeded 1:40 for MN and 1:20 for HI. Age-standardized seroprevalence were calculated using the 2010 National Census population.

**Results:**

Sera from 1,484 individuals were analyzed; 1,363 (92%) were blood donors, and 121 (8%) children or adolescents aged ≤19 years. Mean age (standard deviation) was 31.4 (11.5) years, and 276 (19%) were women. A total of 516 (35%) participants declared history of influenza vaccination after April 2009. The age-standardized seroprevalence to A(H1N1)pdm09 was 48% by the MN and 41% by the HI assays, respectively. The youngest quintile, aged 1 to 22 years, had the highest the seroprevalence; 61% (95% confidence interval [CI]: 56, 66%) for MN, and 56% (95% CI: 51, 62%) for HI.

**Conclusions:**

Despite high transmission of A(H1N1)pdm09 observed immediately after its emergence and extensive vaccination, over a half of the Mexican population remained potentially susceptible to A(H1N1)pdm09 infection. Subsequent influenza seasons with high transmission of A(H1N1)pdm09, as 2011–2012 and 2013–2014, are compatible with these findings.

## Background

The 2009 H1N1 influenza pandemic initially hit Mexico from April 2009 to July 2010. [1] Thirty million doses of the monovalent vaccine against the pandemic H1N1 virus (A(H1N1)pdm09) were delivered to the Mexican population, primarily targeting high-risk groups, from February to August 2010.

Cross-sectional serosurveys of influenza help estimate crude and age-specific prevalence proportions, and assess the probability and size of future epidemics. [2–4] Comparing results across different studies may be challenging as estimates of influenza seroprevalence vary due to dissimilar study populations, time of sera collection, analytical methods, and classification thresholds. [2,5] Seroprevalence estimates may also differ by geographic area even within the same country. To our knowledge, little published information exists on the post-pandemic seroprevalence to A(H1N1)pdm09 virus in Mexico. Only one A(H1N1)pdm09 seroprevalence study measured antibodies by enzyme-linked immunosorbent assay, in late 2009, however the focus was only on persons living in the city of Monterrey, Nuevo León. [6] National public health planning requires assessing susceptibility to infection in representative populations. [7]

To characterize the population immunity to A(H1N1)pdm09 throughout the summer of 2010, we conducted a national serosurvey in six states of Mexico and estimated the proportion of the population with antibodies to influenza A(H1N1)pdm09 as of 2010, using the well-characterized hemagglutination-inhibition assay (HI) and microneutralization assay (MN). [8] To further evaluate the contribution of influenza vaccination to the population immunity, we also collected information on influenza vaccination coverage. One year after the emergence of the pandemic, over half of the Mexican population had anti-influenza antibody titers below the threshold of immunity, rendering them potentially susceptible to A(H1N1)pdm09 infection.

## Methods

### Survey design

To assess the seroprevalence of antibodies to A(H1N1)pdm09 in Mexico, post-pandemic serum samples were collected from 1,484 subjects residing in six states throughout Mexico: Nuevo León and Sonora (in the north), Veracruz (east), Campeche and Chiapas (south), and Mexico City (center). Age of subjects ranged from 1 to 65 years. Sera were collected from June through September 2010, more than a year after the outset of the epidemic and during a vaccination campaign that by October 2010 had delivered 30 million doses of monovalent vaccine against influenza A(H1N1)pdm09, but prior to the 2010/2011 influenza season. The majority of sera (n = 1,363) were collected from adult blood donors. Additional sera (n = 121) were collected from children and adolescents (aged ≤ 19 years) who underwent pre-operatory testing or diagnostic procedures for non-febrile, non-respiratory illness at emergency rooms of public hospitals in Mexico City. Trained personnel of blood banks and participant hospitals collected blood samples using standard procedures.

Study participants were administered a questionnaire to assess their history of acute respiratory illness (i.e., cough, sore throat, and fever), close contact with a person with confirmed influenza, and influenza vaccination, all between April 2009 and the date of the interview. Those who reported receiving influenza vaccination were further asked about the type of vaccine (i.e., the monovalent A(H1N1)pdm09 vaccine, the 2009–2010 trivalent seasonal vaccine for the Northern hemisphere, or both). Participants provided written informed consent themselves or, in the case of children, by their parents. The collection and testing of serum samples at InDRE was considered to be a public health, non-research activity that was exempt from human subject review. Authors only accessed de-identified records from study subjects.

### Titer threshold definition

To determine the sensitivity of the serological assays, paired acute and convalescent (13–75 days after symptom onset) sera from 174 individuals, confirmed to be infected with A(H1N1)pdm2009 virus by real-time reverse-transcription polymerase chain reaction, were tested. Sera were collected from April through July 2009. Participants were residents of Mexico City, Durango, Estado de Mexico, and Queretaro, and their age ranged from 8 months to 75 years. Sensitivity was estimated only on convalescent sera.

To determine the specificity of the serological assays, we analyzed 438 stored serum samples collected between January 2007 thought November 2008 from febrile individuals, aged 0 to 96 years. who tested negative for dengue virus infection. Because the number of individuals over 60 years of age was small, 76 additional samples were obtained from adults aged 60 to 88 years who participated in the Mexican National Health Survey conducted in 2005.

### Laboratory procedures

Sera were analyzed with the MN and HI assays at the Institute of Diagnosis and Epidemiological Reference (InDRE; Mexico City, Mexico) following standard protocols, using an A/California/7/2009-like H1N1 influenza virus, which was propagated in 10 to 11 day-old embryonated chicken eggs. [9]

For the HI assay, sera were treated with receptor-destroying enzyme (RDE; Denke-Seiken, Japan) and, for both assays; sera were heat-inactivated at 56°C for 30 minutes before testing. Sera containing nonspecific agglutinins were pre-adsorbed with turkey erythrocytes before testing by HI. HI testing was performed using 0.5% turkey erythrocytes. Serial two-fold dilutions of serum were tested in duplicate starting at the 1:10 dilution. For both assays, antibody titers were expressed as the reciprocal of the highest dilution of serum resulting in ≥ 50% neutralization or complete hemagglutination inhibition. Titers of <10 were reported as 5.

The geometric mean of the duplicates was considered as the antibody titer for each sample. Thresholds for the MN and HI seropositivity were defined as the titer cut-off points, larger than the limit of detection, that maximize the sum of sensitivity and specificity when testing separate groups of positive and negative sera. Antibody titers of MN ≥ 1:40 or HI ≥ 1:20 were identified as optimal thresholds, with sensitivity of 89% and 63%, and specificity of 85% and 98%, respectively (Table A in S1 File). Pre-pandemic sera were also tested at the Influenza Division, US Centers for Disease Control and Prevention (CDC; Atlanta, Georgia). Both InDRE and CDC reported the same MN and HI titers for an International Standard (IS) for antibody to A(H1N1)pdm09 (09/194) which was tested side-by-side with the sera samples. [10] Therefore, no adjustment of titers was required.

### Statistical analysis

The mean proportion of seropositive individuals was the primary measure of prevalence in the study population. Prevalence was estimated overall and for quintiles of age with limits at: 1 to 22 years (n = 312), 23 to 28 years (n = 309), 29 to 34 years (n = 301), 35 to 41 years (n = 273), and 42 to 65 years (n = 289). We used multivariate log-binomial generalized linear models, with robust standard errors, to estimate prevalence ratios (PR) adjusted by sex, age, self-reported history of vaccination, and self-reported respiratory illness. Age quintiles were included in the model as indicators. Age-standardized seroprevalence for the age range of the survey population (1 to 65 years) was estimated by the direct method using the 2010 Mexican National Census (http://www.inegi.org.mx/est/contenidos/Proyectos/ccpv/) as the reference population. Analyses were conducted in Stata/SE 13.1 for Mac (Stata Corp., College Station, TX).

## Results

### Characteristics of the survey population

A total of 1,484 individuals answered the survey and provided a blood sample for testing; 1,363 (92%) were blood donors and 121 (8%) were children or adolescents aged ≤ 19 years. Mean age (standard deviation) was 31.4 (11.5) years, and 276 (19%) were women. (Table 1)

**Table 1:** Characteristics of 1,484 participants of the influenza A(H1N1)pdm09 serosurvey in blood donors; Mexico, June–September 2010.

Characteristic *	Vaccinated		Unvaccinated		Unknown vaccination status		Total	
	(n = 516; 35%)		(n = 949; 64%)		(n = 19; 1%)		(n = 1,484)	
	Mean, Standard deviation							
Age, years	30.7	12.0	31.9	11.1	25.3	12.9	31.4	11.5
	Number, %							
Women	104	20	166	18	6	32	276	19
State of residency								
Campeche	53	10	66	7	0	0	119	8
Chiapas	76	15	153	16	2	11	231	16
Mexico City	194	38	378	40	12	63	584	39
Nuevo Leon	63	12	82	9	2	11	147	10
Sonora	38	7	112	12	0	0	150	10
Veracruz	92	18	158	17	3	16	253	17
History of respiratory illness †	54	11	56	6	1	5	111	8
Recent exposure to a person with respiratory illness §	10	1	13	3	0	0	23	2

* Percentages may not add up to 100 because of rounding.

† Sixteen individuals who reported unknown recent history of respiratory illness were classified as with no recent respiratory illness. One of these reported recent history of influenza vaccination, two reported unknown history of influenza vaccination, and 13 declared no recent influenza vaccination.

§ Defined as self-reported close contact (i.e., at household) with a person having respiratory symptoms within the two weeks of the survey. A total of 46 individuals that reported unknown contact to a person with respiratory illness were classified as no contact.

### History of influenza vaccination

Of the 1,484 participants, 516 (35%) reported having being vaccinated against influenza between April 2009 and the date of the survey. The proportion of vaccinated individuals varied across states, ranging from 25% in Sonora to 45% in Campeche. (Table 1) Among the vaccinated, 99 (19%) indicated receiving the monovalent A(H1N1)pdm09 vaccine, 167 (32%) the 2009–2010 seasonal trivalent vaccine for the Northern hemisphere, 56 (11%) both vaccines, and 194 (38%) participants did not specify the vaccine type they had received (Table B in S1 File).

The proportion of vaccinated individuals who received the A(H1N1)pdm09 monovalent vaccine ranged from 7% in Chiapas to 38% in Campeche and Nuevo León. (Table B in S1 File). Study participants who received the A(H1N1)pdm09 monovalent vaccine were evenly distributed across age groups. (Table C in S1 File)

### History of acute respiratory illness

A total of 111 (8%) individuals reported history of acute respiratory illness after April 2009. The frequency of self-reported respiratory illness was larger in first age quintile (i.e., 1–22 years) than in all other age quintiles PR: 4.3 (95% confidence interval [CI]: 3.0, 6.1). Compared to those who reported no, or unknown recent vaccination, individuals who reported recent history of influenza vaccination were more likely to also report a history of recent acute respiratory illness; PR: 1.8 (95% CI: 1.2, 2.5).

### Seropositive proportions to A(H1N1)pdm09 in the survey population

Using titer thresholds of MN ≥ 1:40 or HI ≥ 1:20, the crude estimated proportions of seropositives to the A(H1N1)pdm09 virus in the survey population, were 43% (95% CI: 41, 46%) and 35% (95% CI: 32, 37%), respectively. The youngest quintile of age (1–22 years) had the highest proportion of seropositives (61%, 95% CI: 56, 66%; and 56%, 95% CI: 51, 62%) by MN and HI, respectively. (Table 2) Regardless of age, proportions of seropositives were higher in those with self-reported recent history of influenza vaccination than in those without or unknown vaccination history; PR = 2.0 (95% CI: 1.8, 2.3) at MN ≥ 1:40, and 2.2 (95% CI: 1.9, 2.5) at HI ≥ 20. Qualitatively similar results were observed when comparing seropositivity in persons who received monovalent A(H1N1)pdm09 vaccine, alone or with the seasonal trivalent influenza vaccine, to those who did not received either vaccine (Table D in S1 File).

**Table 2:** Number and proportion of the 1,484 participants who tested positive to influenza A(H1N1)pdm09 at the MN ≥ 1:40 and HI ≥ 1:20 thresholds, by quintiles of age.

Age range, years	Number	Age, years		Number and percentage of seropositive individuals *					
				MN ≥ 1:40			HI ≥ 1:20		
		Median	IQR†	n	%	95% CI	n	%	95% CI
1–22	312	19	14, 21	190	61	56, 66	175	56	51, 62
23–28	309	25	24, 27	135	44	38, 49	108	35	30, 40
29–34	301	31	30, 33	106	35	30, 41	76	25	20, 30
35–41	273	38	36, 40	105	39	33, 44	76	28	23, 33
42–65	289	47	44, 51	102	35	30, 41	80	28	23, 33
Overall	1,484	31	24, 39	638	43	41,46	43	35	32, 37

The influenza A(H1N1)pdm09 serosurvey in blood donors; Mexico, June–September 2010.

* Percentages may not add up to 100 because of rounding.

† IQR = interquartile range

### Age-standardized seroprevalence of A(H1N1)pdm09

The 2010 National Population Census estimated 112,336,536 inhabitants in Mexico. Of these, 102,615,001 million persons were aged 1 to 65 years and distributed across the quintiles of the survey population as follows: 47,610,247 million (46%) were aged 1 to 22 years; 11,023,229 million (11%) were aged 23 to 28 years; 10,076,528 million (10%) were aged 29 to 34 years; 11,252,026 million (11%) were aged 35 to 41 years; and 22,652,971 million (22%) were aged 42 to 65 years. The age-standardized seroprevalence to A(H1N1)pdm09 in the census population was 48% for the MN and 41% for the HI assays; which were 12 and 17% higher, respectively, than the corresponding crude seropositive proportions in the survey population. (Table 3)

**Table 3:** Estimated number of seropositive individuals and age-standardized population seroprevalence to influenza A(H1N1)pdm09, at the MN ≥ 1:40 and HI ≥ 1:20 thresholds, for the age range of the survey.

Age range, years*	Number	Population proportion	Estimated number of seropositive individuals in the 2010 census population †		Estimated population seroprevalence, %			
			n		Mean (95% CI)			
			MN ≥ 1:40	HI ≥ 1:20	MN ≥ 1:40		HI ≥ 1:20	
1–22	47,610,247	0.464	28,993,418	26,704,464	61	56, 66	56	51, 62
23–28	11,023,229	0.107	4,815,974	3,852,779	44	38, 49	35	30, 40
29–34	10,076,528	0.098	3,548,545	2,544,240	35	30, 41	25	20, 30
35–41	11,252,026	0.110	4,327,703	3,132,432	39	33, 44	28	22, 33
42–65	22,652,971	0.221	7,995,167	6,270,719	35	30, 41	28	23, 33
Age-standardized	102,615,001	1.000	49,680,804	42,504,632	48		41	

The influenza A(H1N1)pdm09 serosurvey in blood donors; Mexico, June–September 2010.

* Age categories were chosen at the same age limits of quintiles in the study population

† Number of seropositive individuals were back-calculated from the estimated proportions

## Discussion

One year after the emergence of the 2009 H1N1 influenza pandemic and delivery of 30 million doses of the monovalent A(H1N1)pdm09 vaccine, over half of the Mexican population had anti-influenza antibody titers below the threshold of seropositivity used in this study. Children and adolescents had the highest seroprevalence proportion, which is consistent with records of disease surveillance showing this age-group had the highest cumulative incidence of A(H1N1)pdm09 confirmed infection in Mexico within the first year of the emergence of the 2009 influenza pandemic [11,12].

In this study, all 121 children and adolescents (aged ≤ 19 years), who were not blood donors, resided in Mexico City. They represented 8% of the study population, but encompassed 58% of the youngest quintile. Therefore, the highest seroprevalence in this age group may be partially biased by over-representation of some geographic areas in Mexico. Nevertheless, the only previously published influenza serosurvey in Mexico, conducted in a single city (Monterrey, Nuevo León) [6] attained similar findings, as did most serosurveys conducted in other countries from 2010 to 2012. All these studies estimated highest seroprevalence of A(H1N1)pdm2009 in the population aged ≤ 20 years.[4] The skewed age distribution of each study population must be considered when comparing estimates of seroprevalence for influenza A(H1N1)pdm09 across countries. We stratified results by age quintiles to attain enough statistical power for ascertaining age trends in seroprevalence. Seroprevalence proportions for alternative age categories used in other published serosurveys of influenza A(H1N1)pdm09 (Tables E and F in S1 File) showed similar age distribution. Incomplete control of confounding by age may be of concern regardless of the selection of age categories because age does not distribute uniformly within categories. To remove confounding by age, we estimated the age-standardized seroprevalence, which was up to 17% higher than the unadjusted seropositive proportion in the survey population.

Our use of the standard MN and HI assays helps compare seroprevalence estimates to that in other serosurveys. Although an HI titer of ≥ 40 is commonly used as a marker of immunity to A(H1N1)pdm09, [13] our data suggest that this titer threshold may underestimate the number of infected individuals. As in other studies, [9] our HI assay alone was more specific for detecting antibody to A(H1N1)pdm09 while the MN was the most sensitive assay. The combination of threshold titers (MN ≥ 40 or HI ≥ 20) yielded the highest joint sensitivity and specificity to accurately identify individuals who were likely infected by the 2009 virus in the absence of demonstrable seroconversion in paired sera. Despite this, using seropositivity alone may underestimate the true rate of seroconversion because some persons infected with the A(H1N1)pdm09 virus may not develop detectable serological responses, and also because antibody titers elicited by infection or vaccine may subsequently decline below the limit of serological detection. Assuming incomplete detection of seropositive individuals, our findings of detectable antibodies titers in almost half of the population provides a conservative estimate of seroprevalence.

This study encompassed blood donors, who generally are healthy persons between 18 and 65 years of age. Other than the 22 children aged 12 to 36 months, this survey did not include individuals in the high-risk categories defined by the Mexican National Council for Vaccination as targets of the A(H1N1)pdm09 monovalent or seasonal influenza vaccines, in the summer of 2009. Such high-risk groups included: healthy persons aged 6 to 36 months or older than 65 years, pregnant women, the extremely obese; persons with diabetes mellitus, chronic heart or pulmonary disease; and other immunocompromised individuals. More than a third of the participants in all age categories self-reported history of influenza vaccination after April 2009, either with the monovalent A(H1N1)pdm09 or the trivalent seasonal influenza vaccines (Table C in S1 File) indicating that, despite specific high-risk groups were defined in Mexico as targets for vaccination, these vaccines were administered different from planned, reaching also healthy persons of intermediate ages.

Self-reported history of pandemic influenza vaccination was significantly associated with higher age-adjusted seroprevalence, pointing to the expected role of vaccination in increasing immunity to influenza, even one year after the emergence of the epidemic. [12] Persons immunized with the 2009–2010 seasonal trivalent vaccine, which lacked the A(H1N1)pdm09 antigen, also exhibited increased seropositivity to this virus although at significantly lower proportion than those who received the A(H1N1)pdm09 monovalent vaccine. While we cannot rule out that this effect, may in part, be attributed to a misidentification of vaccine type by interviewees, as both vaccines were administered a short time apart, other studies have reported increments in A(H1N1)pdm09 antibody titers in individuals that received previous seasonal influenza vaccines [14,15].

Furthermore, participants of the survey who reported history of acute respiratory illness since the outset of the 2009 pandemic were more likely to be seropositive to influenza A(H1N1)pdm09, but this was unapparent upon adjusting by age. This finding suggests that the purported association of respiratory illness with seropositivity may have resulted from confounding by age as younger individuals, particularly children aged <10 years who have the highest proportions of seropositivity are also known to experience higher frequency of respiratory infections other than influenza. Because both, history of respiratory disease and influenza vaccination were self-reported and collected retrospectively, we cannot rule out the possibility for recall bias.

We found no significant variability of seropositive proportions across the six states of Mexico analyzed in this study, which is also consistent with the findings of surveillance showing that one year after the onset of the influenza epidemic, the A(H1N1)pdm09 virus had reached all regions with similar intensity. [1]

An important limitation of this study is the inclusion of a convenience sample of blood donors and healthy children eligible for surgical procedures, which may not represent the entire Mexican population as it does not include individuals at high risk of influenza. Consequently, the study may have underestimated the seroprevalence. Nonetheless, this study provides evidence that despite the high transmission of A(H1N1)pdm09 observed during the first year of its emergence and extensive vaccination, over a half of the population of Mexico had no or low levels of neutralizing antibodies against A(H1N1)pdm09 virus, rendering them potentially susceptible to A(H1N1)pdm09 infection. Subsequent influenza seasons with high transmission of A(H1N1)pdm09, as 2011–2012 and 2013–2014, are compatible with these findings and further support the value of influenza seroprevalence studies for assessing the risk of influenza transmission. [16,17]

## Supporting Information

S1 File Supplementary tables A to F. (DOCX)

## Abbreviations

A(H1N1)pdm09: Pandemic 2009 influenza virus H1N1
95% CI: 95% Confidence interval
CDC: US Centers for Disease Prevention and Control
HI: Hemagglutination-inhibition assay
InDRE: Institute of Diagnosis and Epidemiological Reference
MN: Microneutralization assay
PR: Prevalence ratio
