# Supplementary material for: A Large Proportion of the Mexican Population Remained Susceptible to A(H1N1)pdm09 Infection One Year after the Emergence of 2009 Influenza Pandemic
Source: PLoS One. 2016 Mar 22;11(3):e0150428. doi: 10.1371/journal.pone.0150428 (PMC4803193; doi:10.1371/journal.pone.0150428)
Supplement: S1 File — (DOCX) [file pone.0150428.s001.docx]

Table A. Sensitivity and specificity of the microneutralization (MN) and hemagglutination inhibition (HI) assays assessed in convalescent sera from 174 PCR-confirmed A(H1N1)pdm09-infected individuals and from 514 individuals unexposed to A(H1N1)pdm09. The influenza A(H1N1)pdm09 serosurvey in blood donors; Mexico, June – September 2010

| Group |  | MN assay with cut-off % (95% CI) | | | | HI assay with cut-off % (95% CI) | | | |
| --- | --- | --- | --- | --- | --- | --- | --- | --- | --- |
|  | N | ≥ 10 | ≥ 20 | **≥ 40** | ≥ 80 | ≥ 10 | **≥ 20** | ≥ 40 | ≥ 80 |
| Sensitivity  (Sera from confirmed cases) | 174 | 90  (85 - 94) | 81  (75 - 87) | **71**  **(65 - 78)** | 55  (47 - 62) | 76  (70 - 83) | **65**  **(58 - 72)** | 49  (41 - 56) | 36  (28 - 42) |
| Specificity  (Pre-pandemic sera) | 514 | 80  (77 - 84) | 91  (89 - 94) | **96**  **(95 - 98)** | 99  (98 - 100) | 94  (92 - 96) | **98**  **(97 - 99)** | 99  (99 - 100) | 100  (99 - 100) |

Table B. Self-reported type of influenza vaccine by state of residency among the 1,484 participants of the influenza A(H1N1)pdm09 serosurvey in blood donors; Mexico, June – September 2010

| State of residency* | n | Type of anti-influenza vaccine †, n (%) | | | | | | | | | |
| --- | --- | --- | --- | --- | --- | --- | --- | --- | --- | --- | --- |
|  |  | Any type | | Monovalent A(H1N1)pdm09 | | Seasonal trivalent§ | | Monovalent and trivalent | | Unknown type | |
| Campeche | 119 | 53 | 45 | 20 | 38 | 16 | 30 | 5 | 9 | 12 | 23 |
| Chiapas | 231 | 76 | 33 | 5 | 7 | 31 | 41 | 11 | 15 | 29 | 38 |
| Mexico City | 584 | 194 | 33 | 28 | 14 | 70 | 36 | 17 | 9 | 79 | 41 |
| Nuevo Leon | 147 | 63 | 43 | 24 | 38 | 7 | 11 | 9 | 14 | 23 | 37 |
| Sonora | 150 | 38 | 25 | 6 | 16 | 15 | 40 | 2 | 5 | 15 | 40 |
| Veracruz | 253 | 92 | 36 | 16 | 17 | 28 | 30 | 12 | 13 | 36 | 39 |
| Overall | 1,484 | 516 | 35 | 99 | 19 | 167 | 32 | 56 | 11 | 194 | 38 |

* Percentages may not add up to 100 because of rounding.

† Nineteen respondents were excluded from analysis because they did not remember whether they had recently been vaccinated against influenza (2 resided in Chiapas, 12 in Mexico City, 2 in Nuevo León, and 3 in Veracruz).

§ The 2009 – 2010 seasonal trivalent influenza vaccine for the Northern Hemisphere included antigens of an A/Brisbane/59/2007 (H1N1)-like virus, an A/Brisbane/10/2007 (H3N2)-like virus, and a B/Brisbane/60/2008-like virus.

Table C. Self-reported type of influenza vaccine by quintiles of age among the 1,484 participants of the influenza A(H1N1)pdm09 serosurvey in blood donors; Mexico, June – September 2010.

| Age rage, years* | n | Type of anti-influenza vaccine †, n (%) | | | | | | | | | | | | |  |
| --- | --- | --- | --- | --- | --- | --- | --- | --- | --- | --- | --- | --- | --- | --- | --- |
|  |  | Any type | | Monovalent A(H1N1)pdm09 | | Seasonal trivalent§ | | | Monovalent and trivalent | | | Unknown type | | |  |
| 1 – 22 | 312 | 114 | 37 | 20 | 18 | | 53 | 47 | | 7 | 6 | | 34 | 30 | |
| 23 – 28 | 309 | 106 | 34 | 20 | 19 | | 30 | 28 | | 12 | 11 | | 44 | 42 | |
| 29 – 34 | 301 | 103 | 34 | 24 | 23 | | 28 | 27 | | 16 | 16 | | 35 | 34 | |
| 35 – 41 | 273 | 99 | 36 | 17 | 17 | | 34 | 34 | | 9 | 9 | | 39 | 39 | |
| 42 – 65 | 289 | 94 | 33 | 18 | 19 | | 22 | 23 | | 12 | 13 | | 42 | 45 | |
| Overall | 1,484 | 516 | 35 | 99 | 19 | | 167 | 32 | | 56 | 11 | | 194 | 38 | |

* Percentages may not add up to 100 because of rounding.

† Nineteen respondents were excluded from analysis because they did not remember whether they had recently been vaccinated against influenza (10 were 1 – 22 years old; 1 was 23 – 28 years old; 4 were 29 – 34 years old; 2 were 35 – 41 years old; and 2 were 42 – 65 years old).

§ The 2009 – 2010 seasonal trivalent influenza vaccine for the Northern Hemisphere included antigens of an A/Brisbane/59/2007 (H1N1)-like virus, an A/Brisbane/10/2007 (H3N2)-like virus, and a B/Brisbane/60/2008-like virus.

Table D. Proportion of seropositive individuals (MN ≥ 1:40 and HI ≥ 20), by state of residency, self-reported history of vaccination and self-reported acute respiratory illness among 1,484 participants of the influenza A(H1N1)pdm09 serosurvey in blood donors; Mexico, June – September 2010.

| Characteristic * | n | Proportion of seropositive individuals, % | | | |
| --- | --- | --- | --- | --- | --- |
|  |  | MN ≥ 1:40 | | HI ≥ 1:20 | |
|  |  | Mean (95% CI) | | | |
| State of residency |  |  |  |  |  |
| Campeche | 119 | 53 | 44, 62 | 41 | 32, 50 |
| Chiapas | 231 | 38 | 31, 44 | 25 | 20, 31 |
| Mexico City | 584 | 45 | 41, 49 | 40 | 36, 44 |
| Nuevo Leon | 147 | 44 | 36, 52 | 36 | 28, 44 |
| Sonora | 150 | 39 | 31, 47 | 32 | 25, 40 |
| Veracruz | 253 | 41 | 35, 47 | 29 | 24, 35 |
|  |  |  |  |  |  |
| Vaccination history † |  |  |  |  |  |
| Monovalent A(H1N1)pdm09 | 99 | 76 | 67, 84 | 72 | 63, 81 |
| Seasonal trivalent | 167 | 58 | 51, 66 | 47 | 39, 54 |
| Monovalent and trivalent | 56 | 73 | 61, 85 | 61 | 48, 74 |
| Unknown type | 194 | 61 | 54, 68 | 50 | 42, 57 |
| None | 949 | 31 | 28, 34 | 24 | 21, 27 |
|  |  |  |  |  |  |
| Acute respiratory illness § |  |  |  |  |  |
| No | 1,373 | 42 | 40, 48 | 34 | 31, 36 |
| Yes | 111 | 53 | 44, 63 | 49 | 39, 58 |

* Percentages may not add up to 100 because of rounding.

† Nineteen respondents were excluded from analysis because they did not remember whether they had recently been vaccinated against influenza (2 resided in Chiapas, 12 in Mexico City, 2 in Nuevo León, and 3 in Veracruz).

§ Other 16 respondents who declared unknown history of recent acute respiratory illness were regarded as with no recent respiratory illness.

Table E. Number and proportion of the 1,484 participants who tested positive to influenza A(H1N1)pdm09 at the MN ≥ 1:40 and HI ≥ 1:20 thresholds, by category of age. The influenza A(H1N1)pdm09 serosurvey in blood donors; Mexico, June – September 2010.

| Age range, years | Number | Age, years | | Number and percentage of seropositive individuals * | | | | | |
| --- | --- | --- | --- | --- | --- | --- | --- | --- | --- |
|  |  |  |  | MN ≥ 1:40 | | | HI ≥ 1:20 | | |
|  |  | Median | IQR† | n | % | 95% CI | n | % | 95% CI |
| 1 – 4 | 34 | 2.5 | 2, 4 | 22 | 65 | 47, 80 | 21 | 62 | 44,79 |
| 5 – 17 | 73 | 13 | 10, 15 | 45 | 62 | 50, 73 | 46 | 63 | 51,74 |
| 18 – 24 | 313 | 21 | 20, 23 | 174 | 56 | 50, 61 | 149 | 48 | 42,53 |
| 25 – 44 | 872 | 33 | 29, 38 | 313 | 38 | 35, 41 | 249 | 29 | 26,32 |
| 45 – 65 | 192 | 50 | 47, 53 | 66 | 34 | 28, 42 | 50 | 26 | 20, 33 |
| Overall | 1,484 | 31 | 24, 39 | 638 | 43 | 41,46 | 515 | 35 | 32, 37 |

* Percentages may not add up to 100 because of rounding.

† IQR = interquartile range

Table F. Estimated number of seropositive individuals and age-standardized population seroprevalence to influenza A(H1N1)pdm09, at the MN ≥ 1:40 and HI ≥ 1:20 thresholds, by age categories for the age range of the survey. The influenza A(H1N1)pdm09 serosurvey in blood donors; Mexico, June – September 2010

| Age range, years | Number | Population proportion | Estimated number of seropositive individuals in the 2010 census population* | | Estimated population seroprevalence, % | | | |
| --- | --- | --- | --- | --- | --- | --- | --- | --- |
|  |  |  | n | | Mean (95% CI) | | | |
|  |  |  | MN ≥ 1:40 | HI ≥ 1:20 | MN ≥ 1:40 | | HI ≥ 1:20 | |
| 1 – 4 | 10,709,951 | 0.104 | 6,929,969 | 6,614,970 | 65 | 47, 80 | 62 | 44,79 |
| 5 – 17 | 26,533,643 | 0.259 | 16,356,355 | 16,719,829 | 62 | 50, 73 | 63 | 51,74 |
| 18 – 24 | 14,207,435 | 0.139 | 7,898,063 | 6,763,284 | 56 | 50, 61 | 48 | 42,53 |
| 25 – 44 | 32,561,188 | 0.317 | 12,359,808 | 9,297,862 | 38 | 35, 41 | 29 | 26,32 |
| 45 – 65 | 18,602,784 | 0.181 | 6,394,707 | 4,844,475 | 34 | 28, 42 | 26 | 20, 33 |
| Age-standardized | 102,615,001 | 1.000 | 49,938,902 | 44,240,420 | 49 |  | 43 |  |

* Number of seropositive individuals in the national population were back-calculated from the proportions estimated in the study sample.
